# Preoperative information needs and preferences of refractive surgery patients: a discrete choice experiment

**DOI:** 10.3389/fmed.2026.1823508

**Published:** 2026-04-13

**Authors:** Jiawen Wei, Yan Jin, Yaxi Chen, Chunyan Song, Xiaoyan Wang, Jinhua Gan, Kunke Li, Juan Yang

**Affiliations:** 1 School of Nursing, Southwest Medical University, Luzhou, Sichuan, China; 2 Shenzhen Eye Hospital, Shenzhen Eye Medical Center, Southern Medical University, Shenzhen, Guangdong, China; 3 The Affiliated Hospital, Southwest Medical University, Luzhou, Sichuan, China

**Keywords:** discrete choice experiment, information needs, preferences, preoperative education, refractive surgery

## Abstract

**Background:**

Individualized preoperative information can enhance patient satisfaction. However, existing studies have methodological limitations, largely adopting a healthcare “supply-side” perspective and lacking a patient-centered approach. Moreover, systematic quantitative assessments of information preferences among refractive surgery patients remain scarce.

**Settings:**

Department of Ophthalmology at a tertiary general hospital in Luzhou and a tertiary ophthalmic specialized hospital in Shenzhen, China.

**Participants:**

A total of 119 patients in the pilot survey phase, and 567 patients in the formal survey phase.

**Methods:**

Attributes and levels were identified through literature review, qualitative interviews, expert panel consultation and importance ranking. An orthogonal design was generated using Ngene for pilot choice sets, with a D-efficient design subsequently optimized for the main survey. Model estimation was performed in Stata 17.0, commencing with a multinomial logit (MNL) model and a random parameters logit (RPL) model to capture unobserved preference heterogeneity. A latent class logit (LCL) model was subsequently estimated to identify preference-based subgroups. Attribute interaction effects were examined to explore potential complementarities and substitutabilities. Finally, Scenario prediction analysis were conducted to predict the uptake probabilities of alternative information packages.

**Results:**

A total of 10,200 choice observations (425 patients) passed the consistency check. Psychological support, mode of information interaction feedback, and duration of information provision were identified as the core driving factors, and two heterogeneous latent subgroups were identified. Joint analysis of attribute interactions showed significant overall effects [*χ*^2^ (21) = 78.17, *p* < 0.001], indicating complex complementary and substitution patterns, though individual interactions were not significant after correction. The optimal combination was identified as “in-depth information” + “standardized + personalized” + “music” + “illustrated manual + video explanation + WeChat push notification” + “real-time interaction” + “one day before surgery” + “<30 min.”

**Conclusion:**

Patients value humanized care, efficient communication, and respect for their time. Clinical education should shift from a “one-size-fits-all” approach to individualized optimization, enhancing overall utility through optimized combinations. The ideal service model constructed in this study provides evidence-based guidance for optimizing preoperative education processes.

## 1 Introduction

Myopia is a major research focus in ophthalmology (1). The global rise in myopia prevalence is largely attributable to the proliferation of digital lifestyles. It is estimated that by 2030, high myopia will affect 517 million people worldwide, or approximately 6.1% of the global population (2). High myopia is not only detrimental to quality of life but is also strongly associated with blinding complications such as myopic maculopathy and retinal detachment (3).

The safety and efficacy of refractive surgery, a well-established medical procedure for correcting visual impairment and improving quality of life (4), have been clinically validated (5, 6). Consequently, it has become an increasingly common elective surgical option. Refractive surgery is elective, non-emergency, and self-paid, and involves invasive procedures on healthy ocular tissue. Comprehensive, comprehensible, and individualized preoperative information is essential for obtaining informed consent, establishing realistic expectations, alleviating anxiety, and enhancing satisfaction (7, 8). It thus represents a critical supporting component for optimizing the clinical management of refractive surgery. Existing studies suffer from methodological limitations in eliciting patients’ information preferences, as they rely primarily on qualitative interviews (9, 10), rating scales (11) or ranking methods (12). These approaches fail to replicate the discrete choice contexts inherent in real-world decision-making. Furthermore, previous research has predominantly adopted a healthcare “supply-side” rather than a patient-centered perspective.

This study is grounded in Random Utility Theory (RUT) and employs a choice-based conjoint analysis (CBC) to design a Discrete Choice Experiment (DCE) (13). As a well-established method for measuring stated preferences, DCE constructs a series of choice sets that simulate real clinical scenarios, requiring respondents to select among competing alternative profiles. This permits inference of the relative importance of each attribute and its levels, along with the trade-off relationships among them (14, 15). This approach advances conventional descriptive surveys and qualitative interviews, which only broadly describe generalized patient concerns. By quantifying the relative importance of each information attribute, identifying preference heterogeneity across distinct patient subgroups, analyzing interactive effects among these attributes, and simulating acceptance probabilities under diverse scenarios, the present study provides rigorous evidence for establishing individualized preoperative information support protocols.

## 2 Methods

### 2.1 Design

The study was conducted in three phases: (i) Attributes and their levels were identified through literature review, qualitative interviews, expert consultation, and importance ranking. (ii) An orthogonal design was generated using Ngene to construct choice sets, which were then administered in a pilot survey. (iii) Informed by the pilot study results, a D-efficient design was employed to construct the final choice sets for the main survey.

### 2.2 Attributes and identification levels

There is no gold standard for attribute selection; however, it must be grounded in thorough preliminary investigation. The selection criteria are: relevance to the research question; practical significance and meaningfulness; clear and unambiguous definition; comprehensive coverage; and quantifiability of both attributes and their levels (16). The number of attributes should not exceed seven (17), and two to four levels per attribute is generally recommended to balance statistical efficiency and cognitive burden (18, 19).

The study initially identified 10 attributes and 24 levels through a literature review. These were then expanded to 11 attributes and 29 levels through qualitative interviews (*n* = 12). Subsequent expert consultation (*n* = 10) revised and refined the attributes. Finally, an importance ranking survey (*n* = 71) was used to prioritize them, ultimately finalizing 7 attributes and 20 levels. As shown in Table 1.

**Table 1:** Final attributes and levels.

Attribute	Levels	Definition
Depth of information content	Basic information	Surgical introduction, preoperative preparation, intraoperative cooperation, postoperative care, psychological care.
	Intermediate information	Based on basic information, including detailed surgical steps, potential risks and complications, and postoperative recovery process.
	In-depth information	Based on intermediate information, including relevant scientific research, expert opinions, and patient case studies.
Mode of information provision	Standardized	Standardized format and content, ensuring accuracy and consistency.
	Standardized + personalized	Based on the standardized content, customized according to the patient’s needs and preferences.
Psychological support	Music	Listening to soothing music for passive relaxation.
	Mindfulness meditation	Guided focused practice for active relaxation
	Stress ball	Squeezing practice provides a physical distraction from tension.
Mode of information delivery	Illustrated manual + video explanation	Information delivered via a combination of an illustrated manual and a video explanation, providing visual and textual content for clarity.
	Illustrated manual + WeChat push notification	Information delivered via an illustrated manual supplemented by regular WeChat push notifications to reinforce key messages.
	Video explanation + WeChat push notification	Information delivered via a video explanation accompanied by regular WeChat push notifications to reinforce key messages.
	Illustrated manual + video explanation + WeChat push notification	Information delivered through an integrated approach combining an illustrated manual, a video explanation, and WeChat push notifications for comprehensive coverage.
Information interaction feedback	Passive feedback	Questions or feedback allowed after explanation, but not required.
	Real-time interaction	Encourages questions and provides immediate feedback to enhance interactivity.
Timing of information acquisition	Day of surgery appointment	The patient receives relevant information on the day of the surgery appointment.
	One day before surgery	The patient receives relevant information 1 day before surgery.
	Three days before surgery	The patient receives relevant information 3 days before surgery.
Duration of information provision	<30 min	Duration of information provision is less than 30 min.
	30 ~ 45 min	Duration of information provision is between 30 and 45 min.
	>45 min	Duration of information provision exceeds 45 min.

### 2.3 Experimental design and questionnaire development

An optimal DCE design should satisfy the principles of level balance, orthogonality, minimal overlap and utility balance (17); however, achieving all these criteria simultaneously is often challenging in practice. This study employed a fractional factorial design (including both orthogonal design and D-efficient design), which is currently one of the most widely used design approaches in discrete choice experiments (20, 21).

The pilot questionnaire consisted of two sections: sociodemographic characteristics and preference measurement tasks. The number of choice tasks was set to be moderate, as an excessive number may lead to respondent fatigue, whereas an insufficient number may reduce statistical efficiency (22). When a large number of choice sets were required, they were blocked into multiple survey versions (17). The design was divided into three blocks (questionnaire versions). Within each block, the frequency of each attribute level was balanced, ensuring that no block disproportionately favored a particular level. Additionally, an opt-out option (e.g., “choose none of these”) was included to reflect real-world decision-making scenarios (16, 19). To further ensure data quality, a duplicate choice task (with the second and ninth choice tasks being identical) was incorporated into the formal survey to perform a consistency check. Responses that failed this check were deemed invalid and excluded from the analysis (23).

### 2.4 Data collection

The study was conducted at the Department of Ophthalmology of a tertiary general hospital in Luzhou City and a tertiary ophthalmic specialized hospital in Shenzhen City from May to November 2025. Inclusion criteria: aged ≥18 years; assessed as suitable for refractive surgery; able to read and comprehend normally; voluntarily participated and provided written informed consent. Sample size estimation was conducted using the Johnson-Orme rule of thumb, one of the most widely cited and accepted standards for sample size calculation in preference research:


$$N\geq \frac{500c}{t\times a}$$

In the formula, 500 represented the numerator constant; N denoted the required minimum sample size; 𝑡 denoted the number of choice tasks per respondent ( t = 8); a denoted the number of alternatives per task, excluding the opt-out option ( a = 2); and c denoted the maximum number of levels across all attributes ( c = 4). Substitution of these values yielded a minimum sample size of 125 respondents per survey version. With three versions, a total of 375 respondents were required. Accounting for an anticipated 20% attrition rate, the target sample size was set at 469 respondents. Data were collected anonymously via the online platform Wenjuanxing.^1^

### 2.5 Statistical analysis

#### 2.5.1 Pilot study phase

The pilot data were analyzed using a random parameters logit (RPL) model in Stata 17.0 to obtain preliminary utility coefficient estimates and their directional signs for each attribute level.

#### 2.5.2 Formal experiment phase

In this study, sociodemographic characteristics were described using frequencies and percentages. In formal experiment phase, preference analysis was performed using a multinomial logit (MNL) model and a RPL model (24). Model statistical performance was evaluated and the best-fitting model was determined using the Log-Likelihood (LL), Akaike Information Criterion (AIC), Bayesian Information Criterion (BIC), and Likelihood Ratio Test (15). A latent class logit (LCL) model was employed to identify sources of preference heterogeneity and latent subgroups (25). Attribute interaction analysis was conducted to examine synergistic and trade-off relationships among attributes (16). Scenario prediction analysis was performed to estimate the choice probabilities of different information packages.

The study defined statistical significance as *p* < 0.05 and considered 0.05 ≤ *p* < 0.10 as marginally significant, in order to address the statistical power challenges in exploring preference heterogeneity within DCEs and to identify potentially meaningful clinical patterns.

## 3 Results

### 3.1 Pilot study phase

#### 3.1.1 Construction of discrete choice sets

Based on the finalized attributes and levels, 36 profiles were generated and paired into six questionnaire versions, each containing six choice sets.

#### 3.1.2 Questionnaire design

The demographic characteristics questionnaire collected information on patients’ age, gender, educational level, employment status, marital status, residence, duration of myopia, severity of myopia, and surgical procedure. A total of 119 participants were enrolled in the pilot study.

### 3.2 Formal experiment phase

#### 3.2.1 Construction of discrete choice sets

Based on the pilot study results (prior coefficients ranging from −0.645 to 0.433; relative D-efficiency value = 0.273, indicating the optimal design among all generated designs), 24 profiles were generated and paired into three questionnaire versions. Each version contained nine choice sets, with the second and ninth choice sets being identical, and included an opt-out option.

#### 3.2.2 Demographic characteristics

A total of 567 patients were included in the discrete choice experiment, of whom 345 (60.85%) were aged 18 ~ 25 years; 334 (58.91%) were male; 466 (82.19%) had received higher education; 441 (77.78%) were unmarried; 411 (72.49%) resided in urban areas; 267 (47.09%) had a myopia duration of 5 ~ 10 years; 325 (57.32%) had moderate myopia; and 433 (76.37%) opted for SMILE surgery. As shown in Table 2.

**Table 2:** Demographic characteristics.

Variable	Category	*N*	Percentage (%)
Age	18–25 years	345	60.85
	26–30 years	107	18.87
	31–35 years	70	12.35
	36–40 years	36	6.35
	>40 years	9	1.59
Gender	Male	334	58.91
	Female	233	41.09
Educational level	Secondary education	101	17.81
	Higher education	466	82.19
Employment status	Employed	279	49.21
	Student	216	38.10
	Unemployed	72	12.70
Marital status	Unmarried	441	77.78
	Married	122	21.52
	Divorced	4	0.71
Residence	Urban	411	72.49
	Suburban	75	13.23
	Rural	81	14.29
Duration of myopia	<5 years	83	14.64
	5 ~ 10 years	267	47.09
	>10 years	217	38.27
Severity of myopia	Mild (−0.50 D to –2.99 D)	122	21.52
	Moderate (−3.00 D to –6.00 D)	325	57.32
	High (< −6.00 D)	120	21.16
Surgical procedure	SMILE	433	76.37
	FS-LASIK	106	18.69
	TransPRK	10	1.76
	ICL	18	3.17

#### 3.2.3 Consistency analysis

As shown in Table 3, the estimation results of the RPL model for both the total sample and the consistent sample are presented. The directions and significance levels of the coefficients were consistent across the two samples. After excluding inconsistent responses, the model fit improved, with the LL increasing from −3,792.8 to −2,793.6, and both the AIC and BIC decreasing by approximately 2,000. Ultimately, 10,200 valid observations (425 respondents) were obtained for analysis.

**Table 3:** Results of consistency check.

Variable	Total sample			Consistent sample		
	Coefficient	SE	*p*	Coefficient	SE	*p*
ASC	−2.774	0.149	0.000	−2.811	0.174	0.000
Intermediate information	−0.125	0.133	0.347	−0.088	0.160	0.579
In-depth information	0.041	0.053	0.442	0.050	0.060	0.406
Standardized + personalized	0.003	0.061	0.949	−0.065	0.085	0.441
Mindfulness meditation	−0.394	0.055	0.000	−0.331	0.062	0.000
Stress ball	−0.234	0.056	0.000	−0.194	0.064	0.002
Illustrated manual + WeChat push notification	0.045	0.068	0.513	0.099	0.080	0.214
Video explanation + WeChat push notification	−0.181	0.070	0.010	−0.090	0.080	0.260
Illustrated manual + Video explanation + WeChat push notification	0.126	0.069	0.067	0.113	0.077	0.142
Real-time interaction	−0.123	0.068	0.069	−0.114	0.081	0.160
One day before surgery	0.102	0.146	0.486	0.097	0.176	0.578
Three days before surgery	−0.090	0.053	0.092	−0.098	0.060	0.104
30 ~ 45 min	−0.170	0.060	0.004	−0.156	0.069	0.024
>45 min	−0.269	0.060	0.000	−0.226	0.070	0.001

Variable	SD	SE	*p*	SD	SE	*p*
ASC	/	/	/	/	/	/
Intermediate information	0.022	0.280	0.935	0.078	0.286	0.784
In-depth information	0.290	0.144	0.043	−0.021	0.202	0.915
Standardized + personalized	0.920	0.088	0.000	1.124	0.136	0.000
Mindfulness meditation	−0.115	0.237	0.625	0.152	0.222	0.492
Stress ball	0.036	0.235	0.877	−0.004	0.183	0.982
Illustrated manual + WeChat push notification	0.019	0.101	0.847	0.007	0.099	0.943
Video explanation + WeChat push notification	0.001	0.134	0.994	0.001	0.144	0.994
Illustrated manual + video explanation + WeChat push notification	−0.355	0.145	0.014	0.010	0.228	0.962
Real-time interaction	1.056	0.091	0.000	1.004	0.129	0.000
One day before surgery	−0.007	0.258	0.978	0.045	0.285	0.872
Three days before surgery	0.235	0.143	0.101	0.042	0.241	0.859
30 ~ 45 min	−0.009	0.142	0.948	−0.019	0.149	0.896
>45 min	−0.370	0.134	0.005	0.430	0.134	0.001
Number of respondents	567			425		
*N*	13,608			10,200		
AIC	7639.5			5641.2		
BIC	7842.5			5836.4		
Prob > chi2	0.0000			0.0000		
log likelihood	−3792.759			−2793.615		

#### 3.2.4 Baseline regression analysis

As shown in Table 4, the baseline regression results of the RPL and MNL models are reported. The coefficient directions were consistent between the two models, indicating good robustness. The RPL model yielded a higher LL value, demonstrating superior model fit; therefore, subsequent analyses were primarily based on the RPL results.

**Table 4:** Baseline regression results.

Variable	MNL			RPL		
	Coefficient	SE	*p*	Coefficient	SE	*p*
ASC	−0.898	0.118	0.000	−2.811	0.174	0.000
Intermediate information	−0.004	0.108	0.965	−0.088	0.160	0.579
In-depth information	0.087	0.048	0.072	0.050	0.060	0.406
Standardized + personalized	0.137	0.040	0.000	−0.065	0.085	0.441
Mindfulness meditation	−0.246	0.052	0.000	−0.331	0.062	0.000
Stress ball	−0.132	0.052	0.011	−0.194	0.064	0.002
Illustrated manual + WeChat push notification	0.078	0.065	0.230	0.099	0.080	0.214
Video explanation + WeChat push notification	−0.062	0.067	0.358	−0.090	0.080	0.260
Illustrated manual + Video explanation + WeChat push notification	0.067	0.062	0.284	0.113	0.077	0.142
Real-time interaction	0.086	0.039	0.029	−0.114	0.081	0.160
One day before surgery	−0.012	0.108	0.909	0.097	0.176	0.578
Three days before surgery	−0.067	0.047	0.156	−0.098	0.060	0.104
30 ~ 45 min	−0.139	0.058	0.016	−0.156	0.069	0.024
>45 min	−0.148	0.054	0.006	−0.226	0.070	0.001

Variable	SD	SE	*p*	SD	SE	*p*
ASC	/	/	/	/	/	/
Intermediate information	/	/	/	0.078	0.286	0.784
In-depth information	/	/	/	−0.021	0.202	0.915
Standardized + personalized	/	/	/	1.124	0.136	0.000
Mindfulness meditation	/	/	/	0.152	0.222	0.492
Stress ball	/	/	/	−0.004	0.183	0.982
Illustrated manual + WeChat push notification	/	/	/	0.007	0.099	0.943
Video explanation + WeChat push notification	/	/	/	0.001	0.144	0.994
Illustrated manual + video explanation + WeChat push notification	/	/	/	0.010	0.228	0.962
Real-time interaction	/	/	/	1.004	0.129	0.000
One day before surgery	/	/	/	0.045	0.285	0.872
Three days before surgery	/	/	/	0.042	0.241	0.859
30 ~ 45 min	/	/	/	−0.019	0.149	0.896
>45 min	/	/	/	0.430	0.134	0.001
*N*	10,200			10,200		
Prob > chi2	0.0000			0.0000		
Log likelihood	−3404.706			−2793.615		

The ASC (coded as 1 for the “opt-out” option and 0 for the improved alternative) had a highly significant negative coefficient of −2.811 in the RPL model, indicating that respondents preferred the improved alternatives. The mode of psychological support, information interaction feedback, and duration of information provision were identified as the core driving factors. In contrast, the depth of information content, mode of information delivery and timing of information acquisition had limited influence. Significant individual preference heterogeneity was observed for the mode of information provision, information interaction feedback, and duration of information provision.

#### 3.2.5 Preference heterogeneity analysis

To determine the optimal number of latent classes, this study fitted models with 2 to 5 classes, using the AIC and BIC as the primary selection criteria. As shown in Table 5. Based on the comparison of the LCL models and the estimation diagnostics, Class 2 was selected as the main focus for analysis and interpretation. First, Class 2 accounted for a significantly larger proportion of the sample (84.3%), representing the dominant preference structure, whereas Class 1 comprised only 15.7%. Second, regarding estimation quality and robustness, when higher numbers of classes (e.g., 3 to 5) were attempted, standard errors for some attribute parameters could not be estimated, suggesting potential class degradation, quasi-complete separation, or identification issues due to insufficient information. In contrast, the key attribute coefficients for Class 2 were statistically significant and stable, with consistent signs, robust estimates, and good interpretability. Furthermore, as the largest and most robustly estimated group, Class 2 provides the most direct evidence for optimizing service strategies. Therefore, considering class size, estimation quality and robustness, as well as applicability, Class 2 was selected as the focal class for interpretation. As shown in Table 6.

**Table 5:** Criteria for selecting the number of classes.

Classes	AIC	BIC
2	5089.063	5206.574
3	5033.667	5211.959
4	4740.964	4980.038
5	4641.709	4941.564

**Table 6:** Latent class model results.

Variable	Class 1			Class 2		
	Coefficient	SE	*p*	Coefficient	SE	*p*
ASC	1.759	0.802	0.028	−3.688	0.278	0.000
Intermediate information	0.146	0.398	0.714	−0.410	0.367	0.264
In-depth information	0.124	0.345	0.719	0.083	0.049	0.090
Standardized + personalized	−0.497	0.304	0.102	0.151	0.040	0.000
Mindfulness meditation	−0.716	0.378	0.058	−0.234	0.054	0.000
Stress ball	0.262	0.323	0.417	−0.150	0.054	0.005
Illustrated manual + WeChat push notification	−0.103	0.398	0.796	0.072	0.068	0.290
Video explanation + WeChat push notification	−0.451	0.416	0.278	−0.058	0.070	0.407
Illustrated manual + video explanation + WeChat push notification	−0.473	0.392	0.227	0.088	0.065	0.176
Real-time interaction	0.199	0.288	0.489	0.080	0.041	0.050
One day before surgery	−0.722	0.394	0.067	0.116	0.412	0.778
Three days before surgery	−0.515	0.349	0.140	−0.064	0.048	0.182
30 ~ 45 min	0.298	0.352	0.397	−0.147	0.061	0.016
>45 min	−0.300	0.380	0.430	−0.142	0.056	0.011
Class membership function						
Age	0.529	0.229	0.029	/	/	/
Gender	0.202	0.328	0.538	/	/	/
Educational level	−1.095	0.347	0.002	/	/	/
Employment status	−0.015	0.233	0.949	/	/	/
Marital status	−1.310	0.555	0.018	/	/	/
Residence	0.172	0.192	0.370	/	/	/
Duration of myopia	−0.180	0.269	0.503	/	/	/
Severity of myopia	−0.355	0.241	0.141	/	/	/
Surgical procedure	−0.176	0.246	0.474	/	/	/
_cons	2.330	1.421	0.101	/	/	/
Class proportions	0.157			0.843		
*N*	10,200					
log likelihood	−2505.478					

Regarding the depth of information content, Class 1 showed positive but non-significant preferences for both intermediate and in-depth information. Class 2 exhibited a positive and marginally significant preference for in-depth information (*β* = 0.083, *p* < 0.10), while their preference for intermediate information was not significant.

Regarding the mode of information provision, Class 1 showed a negative but non-significant preference. Class 2 demonstrated a significant preference for the “Standardized + Personalized” (*β* = 0.151, *p* < 0.01).

Regarding psychological support, relative to “Music,” Class 1 showed a marginally significant negative preference for “Mindfulness meditation” (*β* = −0.716, *p* < 0.10) and a non-significant preference for “Stress ball.” Class 2 exhibited significantly lower utility for both “Mindfulness meditation” (*β* = −0.234, *p* < 0.01) and “Stress ball” (*β* = −0.150, *p* < 0.01).

The mode of information delivery was not significant in either class.

Regarding information interaction feedback, Class 1 showed a positive but non-significant preference, whereas Class 2 demonstrated a significant preference for “Real-time interaction” (*β* = 0.080, *p* < 0.05).

Regarding the timing of information acquisition, Class 1 showed a marginally significant negative preference for receiving information “One day before surgery” (*β* = −0.722, *p* < 0.10), with a non-significant negative coefficient for “Three days before surgery”. In contrast, Class 2 exhibited no significant preferences regarding timing.

Regarding the duration of information provision, preferences in Class 1 were non-significant across all duration levels. In contrast, Class 2 showed significantly negative preferences for both “30 ~ 45 min” (*β* = −0.147, *p* < 0.05) and “>45 min” (*β* = −0.142, *p* < 0.05), consistently indicating their preference for short and efficient information services of less than 30 min.

The membership function coefficients showed how individual characteristics influenced the probability of being assigned to Class 1 (relative to Class 2). Age had a significantly positive effect on the likelihood of belonging to Class 1 (*β* = 0.529, *p* < 0.05), while educational level (*β* = −1.095, *p* < 0.01) and marital status (*β* = −1.310, *p* < 0.05) had significantly negative effects. Gender, employment status, residence, duration of myopia, severity of myopia, and surgical procedure were not statistically significant.

#### 3.2.6 Attribute interaction analysis

To examine the overall effect of the interaction terms, we first performed a joint Wald test for the 21 terms included in the model. The test statistic was *χ*^2^ (21) = 78.17, which was highly significant (*p* < 0.001). Further examination of the individual interaction terms showed that, without adjustment, several interactions—including delivery × time (*β* = 0.131, *p* < 0.05) and delivery × duration (*β* = 0.135, *p* < 0.05)—demonstrated significant complementary effects, whereas others, such as feedback × duration (*β* = −0.341, *p* < 0.10), showed marginally significant substitution effects. However, following Bonferroni correction, none of the interaction terms remained statistically significant (all *p* > 0.05). As shown in Table 7.

**Table 7:** Attribute interaction analysis.

Variable	Coefficient	SE	*p*
ASC	−5.927	2.063	0.004
Depth of information content × mode of information provision	0.180	0.279	0.518
Depth of information content × psychological support	0.071	0.107	0.509
Depth of information content × mode of information delivery	−0.119	0.105	0.259
Depth of information content × information interaction feedback	−0.192	0.225	0.393
Depth of information content × timing of information acquisition	0.269	0.153	0.078
Depth of information content × duration of information provision	0.010	0.122	0.931
Mode of information provision × psychological support	0.257	0.143	0.072
Mode of information provision × mode of information delivery	−0.298	0.153	0.051
Mode of information provision × information interaction feedback	0.315	0.382	0.409
Mode of information provision × timing of information acquisition	0.054	0.268	0.840
Mode of information provision × duration of information provision	−0.393	0.208	0.060
Psychological support × mode of information delivery	0.235	0.086	0.006
Psychological support × information interaction feedback	0.297	0.132	0.024
Psychological support × timing of information acquisition	−0.268	0.101	0.008
Psychological support × duration of information provision	−0.002	0.104	0.979
Mode of information delivery × information interaction feedback	0.228	0.142	0.107
Mode of information delivery × timing of information acquisition	0.131	0.066	0.046
Mode of information delivery × duration of information provision	0.135	0.062	0.028
Information interaction feedback × timing of information acquisition	0.425	0.306	0.165
Information interaction feedback × duration of information provision	−0.341	0.185	0.065
Timing of information acquisition × duration of information provision	−0.124	0.120	0.301
Depth of information content	−0.323	0.720	0.653
Mode of information provision	0.244	1.005	0.807
Psychological support	−1.165	0.378	0.002
Mode of information delivery	−0.621	0.323	0.054
Information interaction feedback	−1.429	0.979	0.144
Timing of information acquisition	−0.887	0.616	0.150
Duration of information provision	0.828	0.530	0.1182

Variable	SD	SE	*p*
Depth of information content	−0.124	0.093	0.183
Mode of information provision	−0.863	0.106	0.000
Psychological support	0.296	0.073	0.000
Mode of information delivery	0.023	0.091	0.796
Information interaction feedback	0.771	0.116	0.000
Timing of information acquisition	0.346	0.058	0.000
Duration of information provision	0.737	0.066	0.000
*N*	10,200		
Prob > chi2	0.0000		
Log likelihood	−2707.250		

Bonferroni correction was applied for 21 interaction tests. After correction, no interaction term remained statistically significant.

#### 3.2.7 Scenario prediction analysis

**Figure 1:**
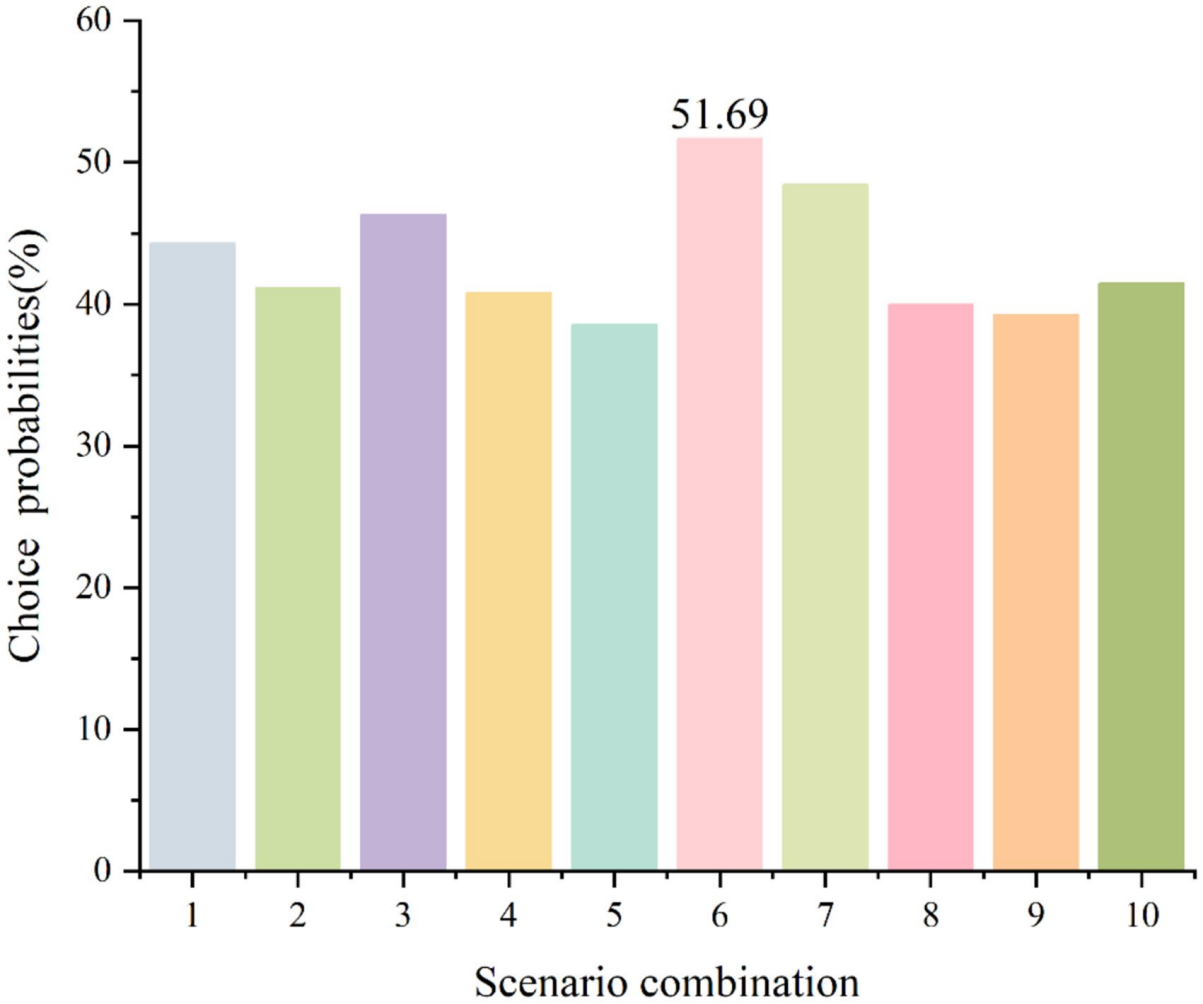
Predicted choice probabilities for different scenario combinations.

Based on model predictions, the choice probabilities of the 10 scenarios varied significantly, ranging from 38.53 to 51.69%. As shown in Table 8, Figure 1. Scenario 6 (“In-depth information” + “Standardized + Personalized” + “Music” + “Illustrated manual + Video explanation + WeChat push notification” + “Real-time interaction” + “One day before surgery” + “<30 min”) achieved the highest choice probability. Compared with baseline Scenario 1, Scenario 2 (replacing “Music” with “Stress ball”) showed a decrease to 41.16%; Scenario 3 (replacing “Passive feedback” with “Real-time interaction”) increased to 46.33%; and Scenario 4 (extending duration to “>45 min”) decreased to 40.79%, providing evidence that psychological support, information interaction feedback, and duration of information provision were key drivers of scenario acceptability.

**Table 8:** Predicted choice probabilities for different scenario combinations.

Scenario	Depth of information content		Mode of information provision	Psychological support		Mode of information delivery			Information interaction feedback	Timing of information acquisition		Duration of information provision		Choice probability (%)
	Intermediate information	In-depth information	Standardized + personalized	Mindfulness meditation	Stress ball	Illustrated manual + WeChat push notification	Video explanation + WeChat push notification	Illustrated manual + video explanation + WeChat push notification	Real-time interaction	One day before surgery	Three days before surgery	30 ~ 45 min	>45 min	
1	0	0	0	0	0	0	0	1	0	1	0	0	0	44.27
2	0	0	0	0	1	0	0	1	0	1	0	0	0	41.16
3	0	0	0	0	0	0	0	1	1	1	0	0	0	46.33
4	0	0	0	0	0	0	0	1	0	1	0	0	1	40.79
5	0	0	0	1	0	0	0	1	0	1	0	0	0	38.53
6	0	1	1	0	0	0	0	1	1	1	0	0	0	51.69
7	0	1	0	0	0	0	0	1	1	1	0	0	0	48.41
8	0	0	0	0	0	0	1	0	0	0	1	0	0	39.96
9	0	0	0	0	0	0	0	0	0	1	0	0	1	39.24
10	0	0	0	0	1	0	0	1	0	0	0	0	0	41.45

The latent class model validated the presence of preference heterogeneity: Class 1, which was more sensitive to psychological support and timing of information acquisition, had the lowest choice probability in Scenario 5 (38.53%), whereas Class 2, which preferred in-depth content, exhibited higher acceptance in Scenario 6 (51.69%).

The interaction effect scenarios further revealed synergistic mechanisms among attributes. The significantly higher attractiveness of Scenario 7, which combined “In-depth information × One day before surgery” and “Music × Real-time interaction”. Although Scenario 10, which included “Stress ball × Day of surgery appointment,” somewhat diminished the advantages of certain attributes, its overall impact remained limited.

## 4 Discussion

### 4.1 Core driving factors

Patients preferred “Music” over “Mindfulness meditation” and “Stress balls,” likely because music support requires neither active engagement nor sustained attentional focus. Multiple systematic reviews have confirmed the positive effects of music support in alleviating perioperative anxiety and pain and improving physiological indicators in patients undergoing ophthalmic surgery (26–29). Significant individual preference heterogeneity was observed for “information interaction feedback”: some respondents preferred “Real-time interaction,” whereas the overall tendency favored “Passive feedback.” This finding may reflect habitual responses shaped by the traditional unidirectional information delivery model in clinical practice. However, in the context of refractive surgery—a procedure characterized by high decision-making involvement—overreliance on Passive feedback may result in information overload (30). Interactive education fulfills patients’ needs for a sense of participation and control through modalities such as educational simulations, educational games, and virtual reality (31). Moreover, a significant negative preference was observed for “duration of information provision,” indicating that patients preferred shorter communication time. This finding suggests that preoperative education should prioritize precision and efficiency over comprehensive coverage to avoid the diminishing marginal utility associated with excessive duration.

### 4.2 Preference of the two groups

Class 1, accounting for 15.7% of the sample and characterized by older age and lower educational level, exhibited a significant negative preference for receiving preoperative education “One day before surgery” and demonstrated a significant need for “Music.” This suggests that a minimal intervention strategy—providing their preferred form of psychological support while avoiding the intervention time point they reject—may enhance satisfaction and acceptance of preoperative care. The ideal preoperative education should begin at the outpatient clinic, continue through pre-admission examinations, and be completed upon formal admission (32). Notably, Lin et al. (33) found that incorporating visual aids into preoperative education for patients with lower educational level undergoing glaucoma surgery significantly enhanced interactivity, comprehensibility, and intraoperative cooperation, while reducing anxiety and pain levels.

Class 2, accounting for 84.3% of the sample and representing the mainstream population, exhibited preferences suggesting a high-quality and efficient service system: they not only sought “In-depth information” but also emphasized a combined “Standardized + Personalized”, along with a “Real-time interaction feedback” mechanism. Notably, while pursuing In-depth information, this group demonstrated high sensitivity to time cost, significantly preferring sessions lasting less than 30 min. For this population, a question prompt list could be developed (34), delivered through multiple interactive modalities. As noted in the editorial by Russell and Exworthy (35), healthcare is witnessing parallel yet seemingly contradictory trends toward both standardization and personalization, necessitating a reconceptualization of how service systems should be designed to align with patients’ needs and expectations. It is essential to ensure that all information is structurally compact and substantively concise, with communication focused on proactive inquiry, avoiding lengthy didactic sessions, and achieving high-quality delivery within 30 min.

These two identified subgroups bridge the gap between the concept of personalized information services and actionable strategies. When resources are limited, nursing staff can use a simple screening tool to classify patients and provide tailored information packages based on their subgroup membership. For example, Chiec et al. (36) developed a Preference-Aligned Communication and Treatment Conversation Trigger Tool to identify patients with cancer in need of goals-of-care conversations. The tool demonstrated good reliability and was clinically well accepted. Minvielle (37) noted that information technology enables large-scale patient classification through the real-time use of massive data at low cost; however, its implementation requires involving end users in the design process and evaluating its acceptability.

### 4.3 Contextual alignment

The effectiveness of a specific mode of information delivery can only be maximized when it is matched with an appropriate duration and timing of receipt. Oexle et al. (38), when comparing different modes of information delivery, did not control for the time participants spent on the content they were assigned, but rather focused on the intrinsic appeal of the different formats themselves. This focus on format alone, however, overlooks a critical dimension of patient decision-making: the demand for cognitive efficiency. Substitution effects capture this dimension, revealing that patients may associate interactive feedback with high efficiency. Once a service combination violates this efficiency contract—for example, by offering interactive features but requiring excessive time—it may trigger resistance, leading to a cancellation of attribute value.

Although individual interaction effects did not reach statistical significance after Bonferroni correction, the joint Wald test showed that the 21 interaction terms were significant as a whole. This overall evidence suggests that the complementary and substitution relationships described above may exist in patients’ preference structures. Nevertheless, individual interaction effects should be interpreted with caution and require further validation in future studies.

### 4.4 Optimal service configuration

Scenario 6 achieved the highest choice probability (51.69%) among the 10 scenarios, emerging as the optimal preoperative information strategy. Regarding content and format, patients desire in-depth information delivered through diverse channels, along with a model that integrates standardization and personalization. Subbaraman et al. (39) evaluated the EyeChoose refractive surgery decision aid. User feedback indicated limitations in information depth, particularly regarding price details, long-term outcome data, and explanations of reversibility mechanisms. Furthermore, the study noted that multimodal information delivery—such as text, embedded multimedia resources, and side-by-side comparisons—can accommodate diverse patient preferences and thereby enhance the overall effectiveness of information transfer. Similarly, large language models have shown promise in supporting ophthalmic patient consultations (40).

In terms of atmosphere and interaction, the proposed scenario integrated music and real-time interaction, further validating the previously identified interaction effect: conducting real-time interaction in an emotionally relaxed setting enhances the patient experience.

In terms of time constraints, this scenario compressed all high-density services into a session of less than 30 min and scheduled it 1 day before surgery. The German Commission of Refractive Surgery recommends providing preoperative information prior to the day of surgery to ensure patients adequate decision-making time (41). From the patient’s perspective, confirming information 1 day before surgery optimally balances information absorption and retention; from a clinical standpoint, it provides a final opportunity to address questions and confirm preparation.

Considering the practical constraints of feasibility and cost-effectiveness, a phased implementation strategy is recommended for Scenario 6. Initially, relatively easy-to-implement components—such as enhancing the quality of information content, incorporating soothing music, optimizing the timing of information acquisition, and shortening the duration of information provision—could be prioritized. For elements that require more substantial technical support or human resources (e.g., personalized services, multimedia integration or real-time interaction), it may be prudent to initiate small-scale pilot programs during non-peak periods (e.g., outside the winter and summer vacation peaks for refractive surgery). Lessons learned from these pilots can then inform broader integration into routine clinical practice.

### 4.5 Limitations

This study employed a cross-sectional design, capturing patient preferences at a single time point. However, as information needs and preferences are dynamic and evolve across the surgical journey, this design could not track their temporal trajectory. Future research could adopt a longitudinal design with multiple waves of discrete choice experiments at key perioperative time points (e.g., preoperatively, 1 week postoperatively, and 1 month postoperatively) to map individual preference trajectories, thereby informing stage-specific targeted education.

In addition, this was a preliminary exploratory investigation conducted across two clinical centers in China. Although the present study enrolled patients undergoing diverse refractive surgical procedures and spanning a range of refractive severities, the overall cohort predominantly consisted of individuals receiving SMILE surgery for moderate myopia. This distribution confers inherent limitations regarding sample representativeness and clinical generalizability. Accordingly, caution is warranted when extrapolating the current findings to broader populations, other surgical subgroups, or diverse geographic settings. Future large-scale, multicenter studies with balanced stratification across all surgical modalities and refractive severity groups will be conducted to further validate and generalize our conclusions.

## 5 Conclusion

As refractive surgery becomes a mainstream option for vision correction, precisely understanding patients’ preoperative information needs is key to advancing patient-centered care. However, existing research has largely adopted a healthcare “supply-side” perspective, offering limited quantitative insights into patients’ true preferences. This study employed a discrete choice experiment to provide empirical evidence on patients’ preference structures, heterogeneity, and the trade-offs they make among multi-attribute information.

The findings confirm that patients value humanistic care, efficient communication, and respect for their time. The identification of two distinct preference subgroups underscores the need to tailor preoperative education to individuals’ cognitive and coping styles, marking a shift from a “one-size-fits-all” approach to individualized optimization. Moreover, the observed complementary and substitution effects among attributes suggest that effective intervention design must prioritize synergistic combinations to avoid utility cancellation from inappropriate pairings. The optimal service configuration identified through scenario simulation offers a concrete, evidence-based benchmark for developing efficient preoperative communication strategies.

## Data Availability

The original contributions presented in the study are included in the article/supplementary material, further inquiries can be directed to the corresponding authors.

## Footnotes

1
https://www.wjx.cn/
